# Gut inflammation and dysbiosis in human motor neuron disease

**DOI:** 10.14814/phy2.13443

**Published:** 2017-09-26

**Authors:** Julie Rowin, Yinglin Xia, Barbara Jung, Jun Sun

**Affiliations:** ^1^ Wellness and Integrative Neurology Westchester Illinois; ^2^ Division of Gastroenterology and Hepatology Department of Medicine University of Illinois at Chicago Chicago Illinois

**Keywords:** ALS, bacteria, dysbiosis, inflammation, microbiome, motor neuron disease

## Abstract

Amyotrophic lateral sclerosis (ALS) is a systemic disorder that involves dysfunction of multiple organs. Growing evidence has shown that neurodegenerative disorders with gut dysbiosis affect the central nervous system via pro‐inflammatory mediators thus impacting gut‐brain communications. We have demonstrated dysbiosis and increased intestinal permeability in the SOD1^G93A^ ALS mouse model. In this study, we comprehensively examined the human gut microbiome in stool samples and evaluated infection and markers of intestinal inflammation in five patients with ALS and motor neuron disorders. Five patients we studied all had alteration in their gut microbiome characterized by a low diversity of the microbiome, compared to healthy cohorts with relatively intact abundance. *Firmicutes* and *Bacteroidetes are* the two major members of bacteria at the phylum level. Low *Ruminococcus spp*. occurred in three patients with low *Firmicutes*/*Bacteroidetes* (F/B) ratio. A majority of patients had signs of intestinal inflammation. This is the first comprehensive examination of inflammatory markers in the stool of ALS patients. Studies in gut health and microbiome related to the onset and progression of ALS may reveal novel therapeutic targets for disease modulation.

## Introduction

The human gut microbiome has been referred to as a “mega organ” harboring 10^14^ microbes and 4 x 10^6^ genes, outnumbering human genes by 150:1 (Bhattacharjee and Lukiw 2013; Collins 2014). Emerging evidence suggests an altered gut microbiome may contribute to the development of neurodegenerative diseases, such as Parkinson's and Alzheimer's disease. The complex interplay of gastrointestinal ‐ central nervous system (CNS) communication involves autonomic and enteric nervous systems, neuroendocrine, and immune systems (Forsyth et al. 2011; Hsiao et al. 2013; Sampson et al. 2016). However, little is known about the intestinal microbiome in patients with amyotrophic lateral sclerosis (ALS) and other motor neuron disorders (MND). ALS is a progressive neurodegenerative disease causing loss of motor neurons in the brain, brainstem, and spinal cord, which leads to loss of voluntary skeletal muscle. The ALS death is due to respiratory failure, typically occurring two to 5 years from diagnosis (Forshew and Bromberg 2003; Miller et al. 2009).

Dysbiosis is defined as an imbalance in the structural and/or functional properties of the gut microbiota. We have demonstrated gut dysbiosis and increased intestinal permeability in the SOD1^G93A^ ALS mouse model (Wu et al. 2015a). SOD1^G93A^ mice harbor the human Cu/Zn superoxide dismutase gene mutation SOD1^G93A^ recapitulating the neuronal and muscle impairment of human ALS. We have shown that using the bacterial product butyrate, a short chain fatty acid (SCFA), improves gut integrity and microbial homeostasis and prolongs lifespan of SOD1^G93A^ mice (Zhang et al. 2017). In this study, we comprehensively examined the gut microbiome and evaluated markers of intestinal inflammation in five patients with MND.

## Methods

### Human subjects

This study was approved by the relevant research ethics committee at the University of Illinois at Chicago. Informed consent was obtained from the research subjects. JR follows patients 1, 2, and 5. Patients 3 and 4 were assessed by release of their medical records and direct email communication (patient 4) or email communication via a first‐hand relative (patient 3) when ALS progression made direct communication difficult. Stool testing was performed on seven samples from five patients. Testing was performed at Genova Diagnostics in four patients (patient No. 2 and No. 5 had repeat confirmatory tests) and at Doctors Data in one patient (patient No. 4). Each laboratory utilizes reference ranges generated from healthy cohorts. For the patient samples tested at Genova Diagnostics, the healthy cohort had 96 individuals recruited with preset exclusion criteria. They did not have intestinal diseases and had no abdominal symptoms. The stool samples of the healthy cohort were collected following the same steps and processed by Genova Diagnostics.

### Stool sample collection

Human stool samples for microbiome test were collected at home using the kit provided by the Genova Diagnostics. Samples were stored at refrigerator before shipping to the company.

### Bacterial diversity and abundance

Commensal Bacteria (PCR) (polymerase chain reaction, Genova Diagnostics) was performed for bacterial imbalance, diversity, and abundance. The 16s rRNA gene sequence (full or partial) for each microorganism was obtained from the NCBI GenBank database. Sequences of related organisms were aligned using Clustal W2 (http://www.clustal.org) to identify conserved regions (for genus probes) and regions of uniqueness (for species probes). Forward (5′–3′) and reverse (3′–5′) primers were chosen based on the level of specificity required of individual assays. The designed primer sequences were verified using NCBI BLAST analysis (http://blast.ncbi.nlm.nih.gov/Blast.cgi), once with the microorganism specified and once with the microorganism excluded from the search to ensure specificity of both the forward and reverse primer sequences. For proprietary right reasons, primer sequences are not shown here.

The relative abundances of commensal bacteria at phylum level from both patients and healthy controls were visualized by abundance bar plots (Xia and Sun, 2017). At the phylum level, bacteria include *Bacteroidetes, Firmicutes, Actinobacteria, Proteobacteria, Euryarchaeota, Fusobacteria*, and Verrucomicrobia. For each phylum, representative bacteria at the class level were tested: *Bacteroides‐Prevotella group, Bacteroides vulgatus, Barnesiella spp., Odoribacter spp.,* and *Prevotella spp*. for *Bacteroidetes; Anaerotruncus colihominis, Butyrivibrio crossotus, Clostridium spp., Coprococcus eutactus, Faecalibacterium prausnitzii, Lactobacillus spp., Pseudoflavonifractor spp., Roseburia spp., Ruminococcus spp*., and *Veillonella spp*. for *Firmicutes. Firmicutes* and *Bacteroidetes are the two major members of bacteria at the* phylum level. *Firmicutes*/*Bacteroidetes (F/B)* ratio was used as an indicator of dysbiosis (Tamboli et al. 2004; Collins 2014; Sampson et al. 2016).

### Infection

Bacterial and mycological culture were performed and observed by microscopy. Bacterial cultures are for *Lactobacillus spp*., *Escherichia coli*,* Bifidobacterium*,* alpha haemolytic Streptococcus, Bacillus species, and gamma haemolytic Streptococcus*. Parasitology EIA Tests were done for Cryptosporidium, Giardia lamblia, and Entamoeba histolytica. No infection by culture was reported at Genova Diagnostics in 4 patients. Yeast culture was tested at Doctors Data. Patient 4 was reported with candida albicans 2 + . Yeast antifungal susceptibility testing was done for patient No. 4.

### Short chain fatty acids in stool samples

MALDI‐TOF MS (Matrix Assisted Laser Desorption Ionization Time‐of Flight Mass Spectrometry) was performed for the concentration of short chain fatty acids (SCFA) in stool samples. Total SCFAs (acetate, n‐butyrate, propionate) reference range >=23.3micromol/g, 29–34.5 is considered borderline low, n‐Butyrate concentration reference range>=3.6 micromol/g.

### Inflammation and immunology

Fecal secretory IgA, calprotectin, and eosinophilic protein X were tested by EIA (enzyme immunoassay) for inflammatory biomarkers.

### Diagnosis and data analysis

For each measurement performed by Genova Diagnostics, the generated value was compared with a reference range (RR) and displayed within a quintile distribution. RRs are usually generated based on two major theories: medical decision points or healthy cohort studies. Some biomarkers, such as calprotectin, are used to differentiate diseases. For those biomarkers, RRs are usually derived from medical literature. If the biomarkers are used to differentiate healthy and sick individuals, RRs are usually generated from studies using healthy cohorts. Results falling outside 2 standard deviations (SDs) from the mean are considered abnormal (see Table**** 1). Results falling outside 1 SD are considered borderline abnormal. It may change depending on if it is one‐tail or two‐tail distribution.

**Table 1:** Patient characteristics and stool analysis results

*N*	Age^a^	Gender	Diagnosis	Stool inflammatory markers/infection	Intestinal microbiome analysis	Short chain fatty acids (SCFA)
1	73	Male	BAD/CLL^b^	Calprotectin **77 mcg/g** f **EPX** c **4.3 mcg/g** **sIgA** e **335 mcg/g** infection‐none	F/B ratio^d^ **5 Low** Diversity **Low** Beta‐glucuronidase 3350 U/g^i^	Total 92.8^g^ n‐Butyrate 11.5^h^
2	82	Female	Bulbar‐onset definite ALS^k^	(Did twice of these tests) Calprotectin **143 mcg/g, 121 mcg/g** EPX 1.6 mcg/g sIgA 208 mcg/g infection‐none	F/B ratio 113 Diversity **Low** Beta‐glucuronidase 591 U/g	Total 69 n‐Butyrate 12.3
3	53	Female	Bulbar‐onset definite ALS	Calprotectin <16 EPX 0.6 **sIgA 449 mcg/g** infection‐none	F/B ratio **9 Low** Diversity **Low** Beta‐glucuronidase **9,991 U/g**	Total 42.4 n‐Butyrate 7.5
4	46	Female	Definite ALS	Calprotectin <10 sIgA 56.8**culture: candida albicans 2+**	Dysbiosis by culture	Total **3.8 (4–18 mg/ml)** n‐butyrate **0.43 Low** **(0.8–4.8 mg/ml)**
5	39	Female	Definite ALS, Celiac disease (confirmed by intestinal biopsy)	(Did twice of these tests ×2) Calprotectin <16 × 2 EPX <DL^j^, 0.5 sIgA 121 mcg/g, 135 mcg/g infection‐none	F/B ratio **18, 10 Low** Diversity **Low × 2** Beta‐glucuronidase 3,737, 3,386 U/g	Total **34.5, 29 Borderline Low** n‐Butyrate 12.2, 8.1 (>=3.6micromol/g)

a In years.

b Brachial Amyotrophic Diplegia/Chronic Lymphocytic Leukemia.

c Eosinophil Protein X (reference range <= 4.6, 4.3 is borderline high).

d Firmicutes/Bacteroidetes ratio (reference range 12–620, 18 is borderline low).

e Fecal secretory IgA (reference range <885 mcg/g, 335 and 449 mcg/g are borderline elevated).

f Calprotectin reference range <50 mcg/g.

g Total SCFAs (acetate, n‐butyrate, propionate) reference range >=23.3micromol/g, 29–34.5 is considered borderline low.

h n‐Butyrate concentration reference range>=3.6 micromol/g.

i Beta‐glucuronidase reference range 368–6266 U/g.

j <DL less than detectable levels.

k Defined by El Escorial Criteria.

The bold fonts are used to shown the changed values.

## Results

### ALS diagnosis and gastrointestinal complaints in patients

We included 5 patients with MND (Table 1) in this study. Patient 1 presented with the bibrachial amyotrophic diplegia variant of ALS and also had chronic lymphocytic leukemia. Patients 2 and 3 presented with bulbar‐onset disease and went on to develop definite ALS (Forshew and Bromberg 2003). Patients 4 and 5 had limb‐onset definite ALS (patient 5 also had celiac disease). Gastrointestinal complaints predated neurological symptoms in all patients. Symptoms included gastroesophageal reflux disease (patient 1, 2, and 4), chronic constipation (patient 1, 2, 3, and 4), intermittent diarrhea (patient 2, 3, 4, and 5) and abdominal pain/bloating (patient 1, 3, and 4).

### Inflammation/Infection

Three of five patients showed inflammatory biomarkers in their stool analyses. All three patients (no 1–3) showed elevated or borderline elevated inflammatory markers (fecal secretory IgA, calprotectin and/or eosinophilic protein X) (Table**** 1). Patient 2 also had non‐specific elevation in calprotectin on two occasions of tests, with specimens collected 3 and 4 months after PEG tube placement. Interestingly, in the two patients lacking inflammatory markers, one had biopsy‐confirmed celiac disease showing moderate to severe villous blunting and increased intraepithelial lymphocytes in the duodenum and had been gluten‐free for 6 and 9 months at the time of stool collection. Patient No. 4 showed infection with 2 +  candida albicans on stool culture.

### Dysbiosis and SCFAs

**Figure 1:**
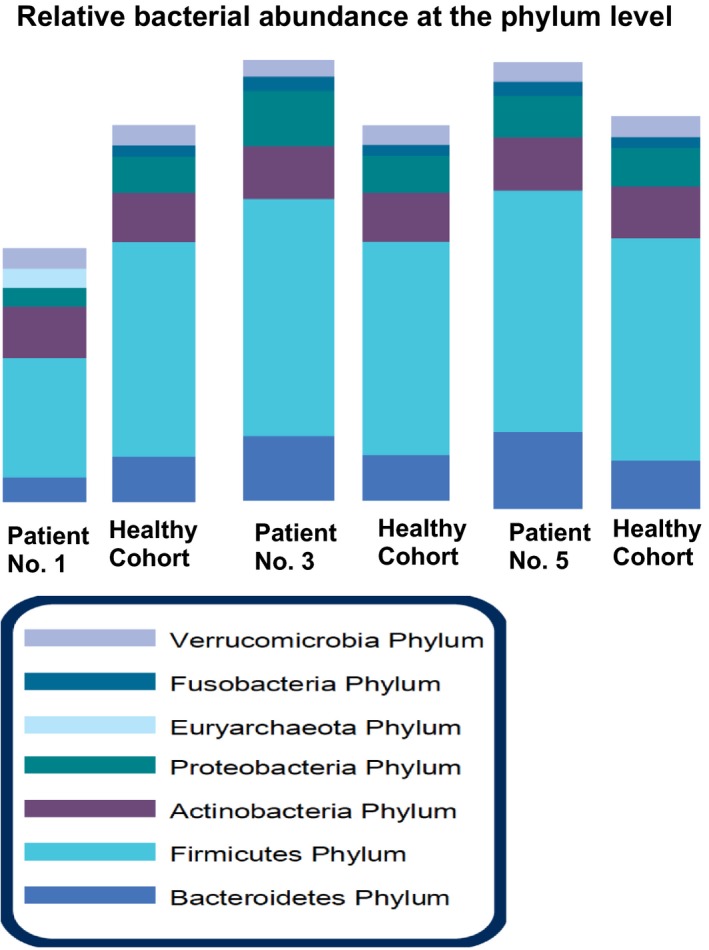
Representative reports of dysbiosis in three Amyotrophic lateral sclerosis (ALS) patients. The relative abundances of commensal bacteria at phylum level from patients and healthy controls were visualized by abundance bar plots. Reduced beneficial bacteria and shift of bacterial profile at the phylum level in ALS patients No. 1, 3 and 5, compared to the health cohort (*n* = 96). *Firmicutes* and *Bacteroidetes are the two major members of bacteria at the* phylum level. *Firmicutes*/*Bacteroidetes (*F/B*)* ratio was used as an indicator of dysbiosis. Three ALS patients all have low F/B ratio.

All patients showed dysbiosis indicated by a decreased diversity of the microbiome compared to healthy cohorts. Patients 1, 3, and 5 had reduced benefit bacteria at the phylum level. As shown in Figure**** 1 with abundance bar plots, patients 1, 3, and 5 all had a low *Firmicutes*/*Bacteroidetes (*F/B*)* ratio, an indicator of dysbiosis. The F/B Ratio was estimated by utilizing the lowest and highest values (12–622) of the reference range for individual organisms. F/B is considered as “low” when the value is less than 12 and 18 is borderline low. In patients 5, two separate tests showed the similar results. Patient 2 did not have a low F/B ratio. This patient was taking probiotic supplements at the time of stool sample collection.

Further analysis of the bacteria at the class level showed that low *Ruminococcus spp*. occurred in all three patients with low F/B ratio. It seems to be a contributor to the low F/B ratio. *Clostridium spp*. and *Roseburia spp*. are low in patient 1. Patient 5 had high *Bacteroides‐Prevotella*,* Odoribacter spp*. *Barnesiella spp*., and *Bacteroides vulgatus*and and patient 3 had high *Bacteroides vulgatus*, which all belong to *Bacteroidetes phylum and* could also contribute to the low F/B ratio.

As shown in Table**** 1 **,** patient 4 had very low levels of total SCFAs and n‐butyrate in the stool samples. Patient 5 had borderline low of total SCFAs.

## Conclusion/Discussion

We have evaluated the gastrointestinal health and stool microbiome profile in a small cohort of patients with motor neuron disorders. All patients had an alteration in their gut microbiome characterized by a low diversity of the microbiome compared to healthy cohorts with relatively intact abundance. A majority of patients had signs of intestinal inflammation. In retrospect, all patients had GI symptoms that predated the onset of neurological symptoms. To the best of our knowledge this is the first comprehensive examination of inflammatory markers in the stool of patients with motor neuron disease. An aberrant microbiome has been reported in prior studies of humans and in the SOD1^G93A^ mouse model in which dysbiosis and a loss of intestinal homeostasis are associated with disease progression (Fang et al. 2016; Zhang et al. 2017). Our current findings add to this line of investigation suggesting a potential role for gut inflammation and microbiome in the development and/or progression of human ALS and MND.

Most intestinal microbial species belong to four major phyla: *Firmicutes*,* Actinobacteria*,* Proteobacteria*, and *Bacteroidetes*. Changes in the ratio of F/B ratio correlate with various human gut disorders, like irritable bowel syndrome (Collins 2014) and inflammatory bowel diseases (Tamboli et al. 2004) and with certain central nervous system disorders like Parkinson's disease (Sampson et al. 2016). Here, we found a low F/B ratio in 3 of the 4 ALS patients. Patient 2 did not have a low F/B ratio. Interestingly, she also had non‐specific elevation in calprotectin on 2 occasions of tests. Calprotectin is released by the gastrointestinal tract in response to infection and mucosal inflammation (Walsham and Sherwood 2016). She was taking probiotic supplements at the time of sample collection. Probiotic supplements have been demonstrated their protective roles in anti‐inflammation and restoring microbiome in various human diseases, including autoimmune diseases (de Oliveira et al. 2017) and multiple sclerosis (Kouchaki et al. 2017). However, it is unknown whether using probiotics in this ALS patient contributes to the current F/B ratio. Unfortunately, we did not have test of her gut microbiome before and after using probiotics.

SCFAs provide energy for the colonocytes and exert beneficial effects in the gut. Butyrate (a SCFA) and butyrate‐producing bacteria are thought to have beneficial effects to the host through anti‐inflammatory properties (Wu et al. 2015b). Along these lines, treatment with butyrate delays disease onset in the SOD1^G93A^ mouse (Zhang et al. 2017). In ALS patients, patient 4 had low butyrate and total SCFA levels in the stool samples and patient 5 had borderline Low of total SCFAs. We cannot draw conclusion that SCFAs are low in human ALS in the current case reports. Our current case report only includes 5 ALS patients. We will need better‐designed experiments and sufficient numbers of patients to study the potential change in SCFAs and dysbiosis in ALS. It will also be interesting to learn the concentration of SCFAs in blood in the future study.

Dysbiosis (e.g., loss of microbial diversity in the gut microbiota) are correlated with intestinal inflammation and change in intestinal integrity (Sun and Chang 2014; Mosca et al. 2016). In the ALS patients we studies, their dysbiosis are correlated with the elevated inflammatory markers (fecal secretory IgA, calprotectin and/or eosinophilic protein X) in stools. Bacterial lipopolysaccharides (LPS), glycolipids found in the outer membrane of gram‐negative bacteria, are pro‐inflammatory and have found to be increased in the serum of ALS patients (Zhang et al. 2009). Indeed, increased inflammatory cytokine IL‐17 and IL‐23 have been reported in serum and cerebrospinal fluid of patients with ALS (Rentzos et al. 2010). We have demonstrated increased serum IL‐17 in the SOD1^G93A^ ALS mice, compared to the wild‐type mice (Wu et al. 2015a). Our study showed the dysbiosis was correlated with intestinal inflammation and increased intestinal permeability in ALS (Wu et al. 2015a). Our current study lacks systemic markers of inflammation. The serum collection will be a next step in a follow‐up study.

ALS is a systemic disorder that involves dysfunction of multiple organs (Toepfer et al. 1999; Forshew and Bromberg 2003; Sun and Zhou 2015). In ALS patients, constipation is common and presumed multifactorial ‐ related to dehydration, lack of dietary fiber intake, and decreased physical activity. However, delayed gastric emptying and delayed colonic transit times have been reported even in the absence of symptoms, suggesting autonomic dysfunction (Forshew and Bromberg 2003). Dysbiosis in neurodegenerative diseases may affect the pro‐inflammatory mediators in CNS and gut‐brain communication via the autonomic, endocrine, and immune systems. It is not clear whether or how intestinal integrity and microbiome are altered in patients before the onset of neurological symptoms. It is reasonable to hypothesize that the human microbiome is an early mediator (Sun and Zhou 2015).

We still lack research on the early intestinal issue from ALS patients. Unfortunately, in the current medical practice, ALS patients are not always asked about their intestinal disease history. Some physicians believe that “early GI complaints are uncommon in ALS patients” and “those patients with GI complaints are not in a typical group of ALS”. We believe that ALS research need team up with multidisciplinary experts and learn from the experience and lessons in the field of inflammatory bowel diseases, obesity, Parkinson's and Alzheimer's disease, multiple sclerosis, and other chronic diseases (Sun 2017). Studies relating the interplay of gut health with the onset and progression of ALS may reveal novel therapeutic targets for disease modulation.

## Ethical Statement

We confirm that we have read the Journal's position on issues involved in ethical publication and affirm that this report is consistent with those guidelines.

## Conflict Of Interest

None of the authors have any conflict of interest to disclose.
